# Supplementary material for: Impact of Abdominal Obesity on Frailty Development: A Web-Based Survey Using a Smartphone Health App
Source: Geriatrics (Basel). 2025 Nov 8;10(6):147. doi: 10.3390/geriatrics10060147 (PMC12641909; doi:10.3390/geriatrics10060147)
Supplement: Supplementary file 1 [file geriatrics-10-00147-s001.zip › Supplementary Table.pdf]

Table S1. The Kihon Checklist.

No.	Questions
1	Do you go out by bus or train by yourself?
2	Do you go shopping to buy daily necessities by yourself?
3	Do you manage your own deposits and savings at the bank?
4	Do you sometimes visit your friends?
5	Do you turn to your family or friends for advice?
6	Do you normally climb stairs without using handrail or wall for support?
7	Do you normally stand up from a chair without any aids?
8	Do you normally walk continuously for 15 minutes?
9	Have you experienced a fall in the past year?
10	Do you have a fear of falling while walking?
11	Have you lost 2 kg or more in the past 6 months?
12	Height: cm, weight: kg, BMI: kg/m^2 If BMI is less than 18.5, this item is scored.
13	Do you have any difficulties eating tough foods compared to 6 months ago?
14	Have you choked on your tea or soup recently?
15	Do you often experience having a dry mouth?
16	Do you go out at least once a week?
17	Do you go out less frequently compared to last year?
18	Do your family or your friends point out your memory loss? e.g.“You ask the same question over and over again.”
19	Do you make a call by looking up phone numbers?
20	Do you find yourself not knowing today’s date?
21	In the last 2 weeks have you felt a lack of fulfilment in your daily life?
22	In the last 2 weeks have you felt a lack of joy when doing the things you used to enjoy?
23	In the last 2 weeks have you felt difficulty in doing what you could do easily before?
24	In the last 2 weeks have you felt helpless?
25	In the last 2 weeks have you felt tired without a reason?

Working Group on Frailty in Japanese Geriatrics Society. BMI, body mass index. Quoted from Arai H and Satake S. English translation of the Kihon Checklist. Geriatr Gerontol Int, 15(4):518-9, 2015.

Table S2. A comparison of the parameters between the included and the excluded respondents aged 30−79 years.

		6,857 respondents aged 30−79 years		p value	95% CI
		3,895 without WC data (excluded)	2,962 with WC data (included)		
Age	years	57.7 ± 10.7	62.7 ± 8.8	< .001	-0.552 — -0.455
Sex	male/female	1405/2490 (36.1%/63.9%)	1250/1712 (42.2%/57.8)	< .001*	---
Number of applicable items in KCL	items/25 items	5.1 ± 3.3	4.6 ± 3.1	< .001	0.260 — 0.569
Exercise habit	yes/no	3052/843 (78.4%/21.6%)	2591/371 (87.5%/12.5%)	< .001*	---

WC, waist circumference; KCL, Kihon Checklist; CI, confidence interval.
Values represent number with percentage or mean ± SD. *: analyzed by chi-square test.

Table S3. Stratified logistic regression analysis according to exercise habit.

Subgroup	Effect of abdominal obesity on frailty development aOR (95%CI)	p value
Exercise habit (yes)	1.259 (0.842–1.883)	0.263
Exercise habit (no)	0.452 (0.141–1.445)	0.181
p for interaction	---	0.030

aOR, adjusted odds ratio; CI, confidence interval.

Adjusted for age, sex, KCL score, frailty awareness, and mean daily steps (covariates from Model 5).

Simple effects represent the association between abdominal obesity and frailty within each exercise subgroup.

p for interaction was obtained from the multiplicative term (abdominal obesity × exercise habit) in the logistic model.
